# Benznidazole Therapy Modulates Interferon-γ and M2 Muscarinic Receptor Autoantibody Responses in *Trypanosoma cruzi*-Infected Children

**DOI:** 10.1371/journal.pone.0027133

**Published:** 2011-10-31

**Authors:** Romina A. Cutrullis, Guillermo F. Moscatelli, Samanta Moroni, Bibiana J. Volta, Rita L. Cardoni, Jaime M. Altcheh, Ricardo S. Corral, Héctor L. Freilij, Patricia B. Petray

**Affiliations:** 1 Servicio de Parasitología y Enfermedad de Chagas, Hospital de Niños Ricardo Gutiérrez, Buenos Aires, Argentina; 2 Instituto Nacional de Parasitología Dr. M. Fatala Chabén, Administración Nacional de Laboratorios e Institutos de Salud Dr. C.G. Malbrán, Buenos Aires, Argentina; National Council of Sciences (CONICET), Argentina

## Abstract

**Objective:**

The presence of autoantibodies with adrenergic and cholinergic activity, capable of triggering neurotransmitter receptor-mediated effects, has been associated with pathogenesis in *T. cruzi-*infected hosts. The goal of this study was to investigate the production of anti-M2 muscarinic receptor autoantibodies (Anti-M2R AAbs) as well as the IFN-γ profile in children at the early stage of Chagas disease, and to examine whether trypanocidal chemotherapy with benznidazole (BZ) could modify both response patterns.

**Methods:**

This study comprised 30 *T. cruzi*-infected children (mean age: 13.8 years) and 19 uninfected controls (mean age: 12.7 years). Infected patients were treated with BZ and followed-up. Blood samples collected at diagnosis-T0, end of treatment-T1, and six months later-T2 were analysed by ELISA for detection of Anti-M2R AAbs and circulating levels of IFN-γ.

**Results:**

At T0, anti-M2R AAbs were demonstrated in 56.7% of *T. cruzi*-infected patients, whereas uninfected controls were 100% negative. The average age of Anti-M2R AAbs^+^ patients was higher than that from negative population. Infected children also displayed significantly stronger serum IFN-γ responses than controls. Upon BZ treatment, a significant linear decreasing trend in Anti-M2R AAb reactivity was recorded throughout the follow-up, with 29.7–88.1% decrease at T2. IFN-γ circulating levels also declined by T2.

**Conclusion:**

Anti-M2R AAbs and IFN-γ raise early during chagasic infection in children and are downmodulated by BZ therapy. These findings reinforce the usefulness of early BZ treatment not only to eliminate the parasite but also to reduce potentially pathogenic immune responses.

## Introduction

Chagas disease, caused by the protozoan parasite *Trypanosoma cruzi,* is one of the worlds leading causes of heart disease [1]. The disease has two successive phases: an initial acute phase lasting between 30 and 60 days and a chronic phase. A majority of the patients that progress to the chronic phase remains clinically asymptomatic for many years, but around 30% of infected individuals progress to disease associated with cardiac and/or digestive disorders [2]. Cardiac manifestations include abnormalities of the intraventricular conduction system, ventricular arrhythmias, sinus node dysfunction, heart failure, aneurysm, and enlargement and dysfunction of the heart [3]. Arrhythmias can be related with cardiomyopathy by itself or with autonomic nervous system alterations [4]. Heart failure and sudden death are the most common causes of decease in patients with Chagas disease [3], [5]. Although clinical signs and symptoms appear several years after infection, pathogenic mechanisms can begin since early stages. Several hypotheses have been proposed to account for pathogenesis in chronic *T. cruzi* infections, including parasite persistence in the myocardium [6], autoimmunity events [7] and tissue injury due to exacerbated inflammatory reactions [8]. Among alterations induced by host self response, there are data supporting autoantibody production, able to interact with β1 adrenergic and muscarinic cholinergic M2 receptors in cardiac tissue [9], leading to early autonomic dysfunction [10], [11]. It has been suggested that the presence of these antibodies could be due to molecular mimicry between human β1 adrenergic/M2 muscarinic receptors and C terminal regions of ribosomal proteins of *T. cruzi* [12]. Recently, this group also demonstrated direct binding of antibodies developed by chronic chagasic patients to the native human β1 adrenergic receptor [13].

Regarding the inflammatory response, several studies have demonstrated that cytokines participate in the control of infection as well as in the induction of pathogenesis during *T. cruzi* infection [14]. Lymphocytes as well as mononuclear cells infiltrating the heart tissue of patients with chagasic cardiomyopathy produce significantly more inflammatory cytokines, such as interferon (IFN)-γ, than blood cells from infected asymptomatic individuals [15]. Moreover, the secretion of IFN-γ has been correlated with the severe cardiac form of Chagas disease [16]. *In vitro* experiments have shown that IFN-γ may induce profound changes in the cardiomyocyte gene expression program, with potential consequences for myocardial contractility, electric conduction and rhythm [17].

*T. cruzi*-infected pediatric patients represent the target population for parasiticidal therapy. Benznidazole (BZ) is the main drug available for the treatment of *T. cruzi* infections. In addition to the direct *in vivo* blocking of parasite growth, BZ-treatment appears to affect host immune regulation [18]. However, little research has been carried out to address the prompt consequences of etiological BZ-treatment on the immune response of children at the early stage of chronic Chagas disease. In this context, we aimed to evaluate the M2 muscarinic receptor autoantibodies (Anti-M2R AAb) response as well as the levels of the proinflammatory cytokine IFN-γ in pediatric patients at the early stage of chronic *T. cruzi* infection. In addition, during the follow-up of BZ-treated patients, we examined whether trypanocidal chemotherapy could modify the patterns of both antibody and cytokine responses.

## Materials and Methods

### Ethics statement

The study protocol was approved by the Research and Teaching Committee and Bioethics Committee of Ricardo Gutiérrez Children's Hospital. Written consent was required from patients' legal representatives, as well as assent from the patient, as appropriate.

### Study population

The prospective follow-up study comprised *T. cruzi*-infected patients admitted to the Parasitology and Chagas Service at Ricardo Gutiérrez Children's Hospital, Buenos Aires, Argentina, for their diagnosis and treatment. Patients with any of the following conditions were excluded: (a) concurrent infections other than *T. cruzi* infection; (b) autoimmune disease; (c) acute or chronic inflammatory process; (d) having received previous etiologic treatment. Age- and sex-matched children seronegative for *T. cruzi* were considered as controls. Our study population (infected patients and uninfected controls) comprised children living in Buenos Aires City and surrounding areas, with similar socioeconomic status. This region is free of vector and is not endemic for Chagas disease, therefore the re-infection is not possible.

### Diagnosis criteria

Serologic diagnosis of *T. cruzi* infection was carried out by indirect hemagglutination (IHA, Lab. Polychaco, Buenos Aires, Argentina), enzyme-linked immunosorbent assay (ELISA, Wiener, Rosario, Argentina) and passive particle agglutination test (PPA, Bayer, Buenos Aires, Argentina).

IHA and PPA antibody titers ≥16, as well as ELISA positive values higher than 1.2, were considered reactive. Infants with at least two positive tests were diagnosed as infected by *T. cruzi*.

### Therapeutic regimen and treatment follow-up

The standard of medical care for *T. cruzi*-infected children is the etiological treatment. Consequently, all infected infants were treated with BZ (RADANIL®, Roche, Buenos Aires, Argentina) at 5–8 mg/kg/day in 2 daily oral doses, for 60 days. Since diagnosis (T0), patients were followed-up by clinical (electrocardiogram-ECG- and echocardiogram-ECHO) and laboratory (IHA and ELISA for *T. cruzi* serology; hemogram, hepatogram, creatinin) evaluations at 30 days, 60 days (end of treatment, T1) and 6 months after completion of chemotherapy (T2). Additionally, parasitological response to treatment was monitored by qualitative polymerase chain reaction (PCR) to detect the presence of *T. cruzi* DNA in blood samples [19]. Ethical considerations precluded the inclusion of an untreated control group in the study taking into account that immediate administration of benznidazole is crucial to achieve therapeutic success.

### Determination of anti-autonomic neurotransmitter antibodies

Serum samples obtained at different times (T0, T1 and T2) of follow-up were stored at −20°C until measuring anti-M2R IgG autoantibody levels by a commercial ELISA kit according to manufactureŕs instructions (Chagacor, Lab. Lemos S.R.L, Buenos Aires, Argentina).

### IFN-γ measurements

Individual IFN-γ serum levels were quantified by double sandwich ELISA (OptEIA Kit for IFN-γ, BD PharMingen, San Jose, CA, USA) according to manufacturer's instructions. Supplied standards were used to generate a standard curve. The assay sensitivity was 2 ng/ml.

### Statistical analysis

Data are expressed as mean value ± standard error of the mean (SEM) or median value (interquartile range). IHA titers were log _2_-transformed before applying the statistical test. ELISA values were expressed as the ratio (R) between optical densities (OD) determined for each specimen and cut-off value. To compare two groups, Student *t* test or Mann-Whitney test were used. One-way analysis of variance (ANOVA) followed by the Tukey test or Friedman test was applied to evaluate changes before and after treatment. Fisher's exact test was used to compare between-group proportions. *P* values of <0.05 were considered significant. Statistical analyses were carried out using the Prisma 4.0 software (GraphPad, San Diego, CA, USA).

## Results

### Patients' characteristics

A total of 30 *T. cruzi*-infected patients were enrolled in this study. The mean age was 13.8 years (range, 8–17 years) and female/male ratio was 1 (15 female *vs* 15 male). On the admission, all of them presented positive serology for Chagas disease at least by 2 conventional assays. Anti-*T. cruzi* antibody titers measured by ELISA and IHA tests were 8.8±0.5_and 6.5±0.2, respectively. All children were asymptomatic and without cardiac compromise (normal ECG and ECHO). Route of infection was transplacental in 27% of patients, vectorial in 4.5% and undefined in 68.5%. Maternal origin of congenitally infected children was Argentina in 46% of cases, Bolivia in 46% and Paraguay in 8%. The control group included 19 uninfected children whose mean age was 12.7 years (range, 7–17 years) and female/male ratio was 1.4 (11 female *vs* 8 male). There were no significant differences in the age and sex distribution between *T. cruzi*-infected children and uninfected controls.

### Pre-treatment Anti-M2R AAb and IFN-γ responses

**Figure 1:**
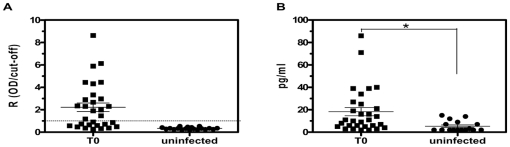
Anti-M2R AAb and IFN-γ profiles in pediatric chagasic patients at T0 and uninfected controls. (A) Distribution of anti-M2R AAb in sera. The group mean and the standard error of the mean are depicted. Dotted line shows cut-off level of the assay. (B) Circulating levels of IFN-γ. The group mean and the standard error of the mean are depicted. * *p* = 0.015 compared to uninfected controls.

Children were examined for the presence of serum Anti-M2R AAb and the results are shown in Figure 1A. AntiM2R AAb reactivity was verified in 56.7% of *T. cruzi*-infected patients, whereas uninfected controls were 100% negative (*p* = 0.0001). The R value (mean ± SEM) in *T. cruzi*-infected patients was 2.2±0.4. The average age of Anti-M2R AAb^+^ patients was significantly higher than that from negative population: 14.6±0.6 years and 12±0.8 years, respectively (*p* = 0.0038). *T. cruzi*-infected children also displayed higher serum IFN-γ levels compared to uninfected controls (*p* = 0.015) (Figure 1B).

### Modulation of Anti-M2R AAb and IFN-γ responses after BZ therapy

**Figure 2:**
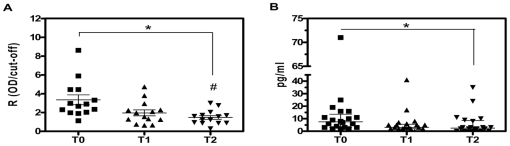
Anti-M2R AAb and IFN-γ profiles in pediatric chagasic patients before and after BZ treatment. (A) Distribution of anti-M2R AAb in sera. Each point represents one patient positive for anti-M2R AAb. The group mean and the standard error of the mean are also depicted. * *p* = 0.001, test for linear trend. # *p*<0.01, T2 *vs* T0. (B) Circulating levels of IFN-γ. Horizontal lines show median values. * *p*<0.05, T0 *vs* T2.

In the course of BZ treatment, ten children withdrew from the study due to adverse events (n = 2), moving to another city (n = 4) or not returning (n = 4). Among Anti-M2R AAb^+^ children at T0, we verified a significant (*p* = 0.001) linear decreasing trend in their autoantibody titers throughout the follow-up (Figure 2A). At T2, ELISA values were significantly lower (*p*<0.01) than those detected at T0, representing a decline ranging between 29.7 and 88.1% of the initial level. No correlation between autoantibody response and heart dysfunction could be established in pediatric chagasic patients, as all children presented normal ECG and ECHO examinations.

Serum concentration of IFN-γ was also determined as shown in Table 1 and Figure 2B. The median value of IFNγ at T2 was significantly lower that that observed at T0 (2.5 pg/ml *vs* 7.5 pg/ml, *p*<0.05). Results for these T0 *vs* T2 pairs of sera revealed a drop in IFN-γ levels that ranged between 10 and 100% of the initial value. No significant differences were observed in the comparison of IFN-γ levels at T0 *vs* T1. We were not able to verify any significant correlation between Anti-M2R AAb and IFN-γ levels. The decrease in Anti-M2R AAb and IFN-γ responses in the lapse between T0 and T2 was accompanied by the negativization of PCR for *T. cruzi* in 94% of chagasic children, showing a rapid decrease in parasite load after treatment.

**Table 1:** Distribution of IFN-γ values for *T. cruzi*-infected and uninfected children.

	Median (pg/ml)	P25	P75
**Infected**			
T0	7.5	3	13.5
T1	3	2	5.5
T2	2.5 *	1	8.5
**Uninfected**	2	2	8

P25: 25^th^ percentile. P75: 75^th^ percentile. * *p*<0.05, T2 *vs* T0.

Changes in anti-*T. cruzi* antibody levels after BZ therapy were also evaluated. However, the analysis of the pairs of sera carried out at T0 and T2 did not show a statistically significant difference: ELISA titers were 8.8±0.5 *vs* 7.7±0.7, respectively (*p* = 0.08) and IHA titers were 6.5±0.2 *vs* 6.2±0.3, respectively (*p* = 0.28). Given that the ascertainment of resolution of *T. cruzi* infection is based upon specific serology reverting to negative, this remaining antibody reactivity prevented the confirmation of cure of treated pediatric patients at the end of the 6-month follow-up.

## Discussion

Autoantibodies with adrenergic and cholinergic activity, capable of triggering neurotransmitter receptor-mediated effects, have been detected in human [20] and murine [21] chagasic sera. Although further *in vivo* experiments are necessary to reliably establish the contribution of these antibodies to *T. cruzi*-mediated cardiomyopathy [22], they may promote dysautonomia often found in chronic Chagas patients [23]. Indeed, this kind of autoantibodies are observed with higher frequency in adults with chronic Chagas heart disease than in asymptomatic *T. cruzi*-infected individuals, suggesting that self-reactive antibodies could be used as a premature marker of evolution for Chagas cardiomyopathy [24]–[26]. In a preliminary report, we demonstrated for the first time the induction of Anti-M2R AAb in pediatric patients at early stages of chronic *T. cruzi* infection [27]. In addition, a clearly decreasing trend in Anti-M2R AAb reactivity down to healthy levels, accompanied by negative conversion of *T. cruzi* serology, was verified by 18–36 months after trypanocidal chemotherapy.

In the present work, an extension of our previous study, we observed a significant reduction in Anti-M2R AAb titers as early as 6 months after completing BZ treatment. Noticeably, the mean age of Anti-M2R AAb^+^ chagasic children was higher than that recorded for the negative population, further emphasizing the need for early trypanocidal therapy in *T. cruzi*-infected young individuals. Taken together, our findings indicate that in pediatric Chagas patients Anti-M2R AAb may be downregulated promptly upon BZ administration.

In some *T. cruzi*-infected children, pretreatment Anti-M2R AAbs levels were similar to those found in uninfected controls. Whether these patients have better prognosis remains to be elucidated. On the other hand, a subgroup of infected children presented high Anti-M2R AAb reactivity with potential pathogenic consequences. The described association between Anti-M2R AAb and cardiomyocyte dysfunction could be coupled to a putative agonistic activity of anti-muscarinic receptor antibodies, capable of inducing a receptor desensitization phenomenon [28]. It has been reported that adult patients at the early stages of Chagas disease have circulating Anti-M2R AAb linked to abnormal modulation of vagal activity [11]. Furthermore, administration of BZ during the advanced phase of experimental *T. cruzi* infection prevented the development of a more severe form of chronic cardiomyopathy, concomitantly with a decrease in the cardiac M2R-specific antibody response [21].

We searched for any correlation between anti-cardiac autoantibodies and heart dysfunction in our pediatric population with *T. cruzi* infection. In contrast with the findings of another research group who found a high frequency of early electrocardiographic alterations in seropositive children from endemic areas [29], we were unable to detect ECG or ECHO abnormalities. This discrepancy might be attributed to the size and characteristics of the populations evaluated for each study. Unlike our report, de Andrade and co-workers [29] examined a large population of seropositive children from a Chagas-endemic area of central Brazil, where the probability of reinfection, as well as increased pathogenicity of infecting strains of *T. cruzi* prevalent in the zone, could account for the differences observed in ECG profiles. Because antibodies that bind to myocardial cholinergic receptors in patients with Chagas disease have shown to trigger physiological, morphological, enzymatic and molecular alterations [30], it is conceivable that these abnormalities could start from the beginning of *T. cruzi* infection even in asymptomatic individuals with normal ECG and ECHO [26], [11].

Clinical and experimental evidences support the involvement of inflammatory cytokines in the pathogenesis of myocardial dysfunction. In this regard, IFN-γ released by infiltrating lymphocytes, both systemically and locally, has been shown to contribute to the impaired cardiac function in the experimental model of autoimmune myocarditis [31]. We herein evaluated the impact of BZ therapy on expression of the inflammatory cytokine IFN-γ in *T. cruzi*-infected children. IFN-γ levels were found to be elevated in *T. cruzi*-infected pediatric patients before initiation of BZ treatment. In agreement with our results, previous studies demonstrated the expression of IFN-γ in young chagasic patients from an endemic region of Paraguay, during the acute and indeterminate stages of infection [32]. In our study population, IFN-γ levels decreased promptly between the time of diagnosis and 6 months after BZ treatment. These findings could well be correlated with the decline in the frequency of peripheral IFN-γ-producing T cells specific for *T. cruzi* observed as early as 12 months after the administration of BZ to chronically infected adults [33]. An exacerbate production of IFN-γ may favour the cytolytic effects of lymphocytes during Chagas disease increasing the chances of myocellular destruction in heart tissue [34]. Consequently, our observations highlight the usefulness of the immunomodulating ability of BZ therapy in *T. cruzi* infection, where a disbalanced inflammatory response becomes detrimental.

Interestingly, IFN-γ was also associated with a muscarinic receptor-mediated decrease in heart contractility [31]. On the other hand, experimental studies suggested that IFN-γ binding to its specific receptors in the heart may lead to a cholinergic response by interaction of both receptor systems on the surface of atrial cells [35]. Our results indicate that treatment with BZ in the early chronic phase of infection could help prevent the development of immune mechanisms involved in the genesis of severe chronic cardiomyopathy, despite the lack of complete parasite eradication [21]. Although the mechanism by which BZ modulates the production of Anti-M2R AAb and the expression of IFN-γ is unclear, the immunomodulating influence of BZ has been widely documented. Parasiticidal chemotherapy proved capable of modulating phagocyte activation, T-cell profile and the release of immune response mediators, like nitric oxide and cytokines [36]. Accordingly, in pediatric patients acutely infected with *T. cruzi*, elevated circulating levels of CD8 and IL-2 soluble receptors were detected, which decrease after receiving BZ therapy [37]. Whether the impact of BZ treatment on both Anti-M2R AAb and IFN-γ responses is a result of parasite load reduction or a direct effect of the drug on the immune system, is still an open question.

The so far accepted criterion of cure for Chagas disease relies on negative seroconversion of the humoral anti-*T. cruzi* response after etiological treatment. Nevertheless, in pediatric patients initiating therapy at the early chronic stage of infection, negative seroconversion occurs several years after treatment [38], therefore requiring long-term follow-up. Our present findings suggest that the BZ-dependent decline of Anti-M2R AAb and IFN-γ levels might provide a surrogate indicator of early therapeutic success in *T. cruzi*-infected children. Further studies on a larger number of infected individuals are needed to clarify their potential use as novel biomarkers of disease prognosis for those chagasic patients who test positive for Anti-M2R AAb and/or IFN-γ before the onset of treatment.

In conclusion, in pediatric patients the Anti-M2R AAb and the IFN-γ responses are elicited early in the course of *T. cruzi* infection, and decrease after trypanocidal therapy with BZ, even though the negative seroconversion of *T. cruzi*- specific antibodies is not fully accomplished. These findings reinforce the usefulness of BZ treatment and encourage further studies to elucidate the potential use of these immune mediators as biomarkers of disease prognosis.
